# Centipede Venom: Recent Discoveries and Current State of Knowledge

**DOI:** 10.3390/toxins7030679

**Published:** 2015-02-25

**Authors:** Eivind A. B. Undheim, Bryan G. Fry, Glenn F. King

**Affiliations:** 1 Institute for Molecular Bioscience, the University of Queensland, St Lucia, Queensland 4072, Australia; E-Mail: g.king@imb.uq.edu.au; 2 School of Biological Sciences, the University of Queensland, St Lucia, Queensland 4072, Australia; E-Mail: bgfry@uq.edu.au

**Keywords:** centipede venom, toxins, evolution, pharmacology, envenomation

## Abstract

Centipedes are among the oldest extant venomous predators on the planet. Armed with a pair of modified, venom-bearing limbs, they are an important group of predatory arthropods and are infamous for their ability to deliver painful stings. Despite this, very little is known about centipede venom and its composition. Advances in analytical tools, however, have recently provided the first detailed insights into the composition and evolution of centipede venoms. This has revealed that centipede venom proteins are highly diverse, with 61 phylogenetically distinct venom protein and peptide families. A number of these have been convergently recruited into the venoms of other animals, providing valuable information on potential underlying causes of the occasionally serious complications arising from human centipede envenomations. However, the majority of venom protein and peptide families bear no resemblance to any characterised protein or peptide family, highlighting the novelty of centipede venoms. This review highlights recent discoveries and summarises the current state of knowledge on the fascinating venom system of centipedes.

## 1. Introduction

**Figure 1:**
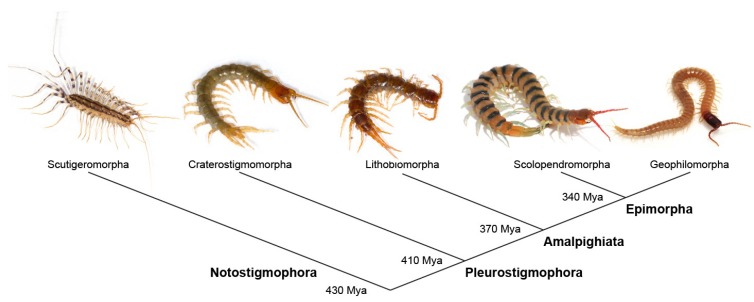
Phylogenetic relationship between the five extant centipede orders according to the Amalpighiata hypothesis. Times since divergence are based on Fernández *et al.* [3].

Class Chilopoda, or centipedes, represents one of the four major myriapod lineages (Arthropoda; Myriapoda). They are present on every continent except Antarctica and are an important group of terrestrial predatory arthropods. There are about 3500 species worldwide within five extant orders: Scutigeromorpha (“house centipedes”), Lithobiomorpha (“stone centipedes”), Craterostigmomorpha (only two congeneric species), Geophilomorpha (“earth centipedes”), and Scolopendromorpha (the largest, most commonly media-documented centipedes) (Figure 1). Several morphological characters unite the members of Chilopoda, of which the most obvious is the modification of the first pair of walking legs into venomous appendages known as poison claws, toxicognaths, maxillipeds, or more correctly forcipules [1]. These are used to capture a wide variety of prey, including insects, spiders, crustaceans, snails, amphibians, reptiles, and even mammals; scutigeromorphs feed primarily by ambushing and chasing down prey, while the other orders seem to rely on opportunistic encounters [2].

Centipedes are thought to have split from the remaining myriapods at least 460 million years ago (mya) [3]. The oldest recognizable order from the fossil record is Scutigeromorpha, of which fossilized legs belonging to a *Crussolum* sp. have been found from the late Silurian almost 420 mya [4]. The earliest fossilized forcipules, from the early Devonian about 400 mya, belonged to the same genus *Crussolum* and are similar to those of the modern scutigeromorph *Scutigera coleoptrata* [5]. The centipede venom apparatus had evolved well before this, however, since the basal split within Chilopoda between Notostigmophora (Scutigeromorpha) and Pleurostigmophora (remaining orders) occurred approximately 430 mya [3]. The centipede venom apparatus thus represents one of the oldest extant venom systems known among terrestrial animals, probably even preceding evolution of the venom systems of scorpions and spiders [6,7].

Unlike scorpions and spiders, centipede venoms have attracted relatively little attention, partly due to their cryptic nature and generally small body size and in part due to their lack of medical importance. Venom extraction in centipedes can be time-consuming, and venom yields are typically very low; even relatively large centipedes such as *Scolopendra polymorpha* (~10 cm) and *S. subspinipes* (~15 cm) yield an average of 1.1 and 5 µL of venom, respectively, when milked using electrostimulation [8]. However, recent advances in the analytical methods employed in toxinological studies have enabled broader study and appreciation of venomous animal diversity, including more challenging taxa such as centipedes [9]. Consequently, a number of substantial discoveries and advances in the fields of centipede toxinology and centipede venom-based biodiscovery have been made since the first review on centipede venoms in 2011 [2]. This review therefore aims to summarize current knowledge on centipede venoms and provide an updated nomenclatorial framework for organisation and naming of centipede toxins.

## 2. Venom Apparatus

Centipede forcipules are shaped like a set of piercing forceps, each consisting of four or five segments: a large trochanteroprefemur, two short segments (femur and tibia), and an apical claw. While the apical claw is made up of two segments in Scutigeromorpha, the tarsus and ungulum, these are fused in all other centipedes and hence referred to as the tarsungulum [1]. The outer surface of each claw contains at least three types of sensilla ceoloconica-type chemoreceptors, which may be used for tasting prey, stimulating the secretion of venom by sensing penetration by the apical claw, or both [10,11]. Interestingly, the evolutionary progression from walking appendages to highly specialised venom delivery systems can be traced by comparison of forcipules from extant centipede orders [12]. This reveals a gradual transformation of the plesiomorphic, slender forcipules found in Scutigeromorpha to the highly modified forcipules found in Geophilomorpha.

The venom glands of most centipedes are pear-shaped, with the exception of scolopendrid centipedes where they are elongated and kidney-shaped. The proximal segments of the forcipules usually contain the venom gland, which line the cuticle along the outer curvature of the appendage and terminate near the base of the forcipule. There are, however, some interesting exceptions. Within the genus *Cryptops* (Cryptopidae, Scolopendromorpha), for example, glands can vary from pear-shaped organs occupying a significant volume of the forcipule to just a few glandular cells [13,14,15]. Gland size also varies within the Scolopendridae, such as in *Asanada socotrana* and *Arthrorhabdus formosus* where they extend into the posterior part of the forcipular coxosternite [16]. The most extreme variation, however, can be found among geophilomorph centipedes. In *Henia vesuviana* (Dignathodontidae), the venom glands are located in the trunk, between the 12th and 18th segments, while in *Aphilodon angustatus* (Aphilodontidae) these are placed even further back into the trunk, between the 15th and 23rd segments [2]. In the latter case, each gland is placed in front of the other and even occupies most of the volume of the three segments it spans [17]. 

While the forcipules are modified walking appendages, the venom gland is thought to have evolved through invagination of the cuticle and weaponization of the cuticular dermal glands [2,18,19,20]. This is evident from the chitinous duct, and the observation that the venom gland is actually a composite glandular epidermis composed of discrete sub-glands, or secretory units. Each secretory unit includes a distal and a proximal canal cell, one or more secretory cells, and an intermediate cell that line an extracellular storage space. These secretory units are individually connected to the lumen through a one-way valve formed by the distal canal cell that penetrates the chitinous duct though a pore. Venom is then expelled from the porous region of the duct, known as the calyx, and through the distal non-porous duct that terminates as a pore (“meatus”) located on the outer curvature near the tip of each claw [1,20].

## 3. Molecular and Pharmacological Diversity

**Figure 2:**
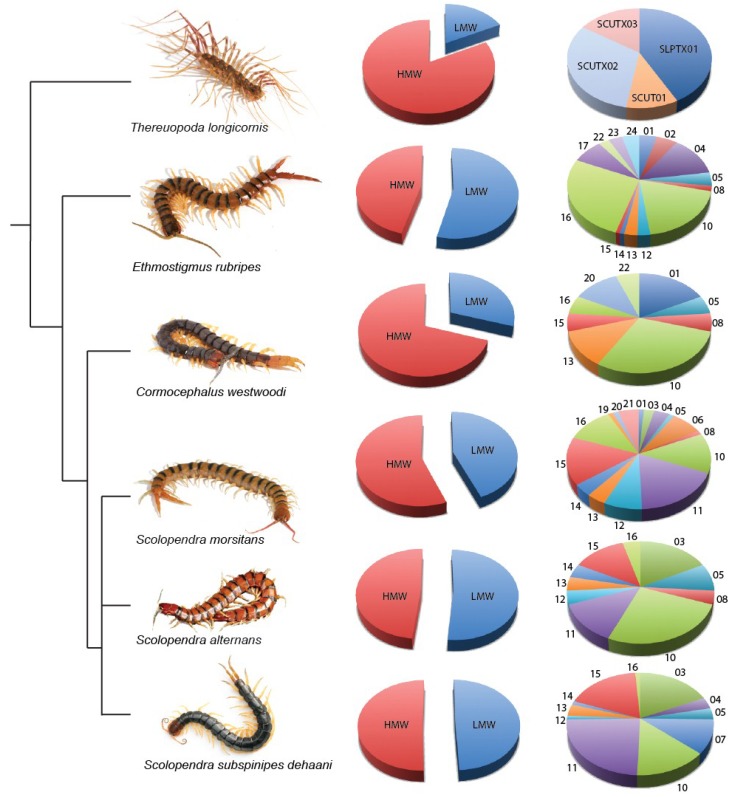
Representative phylogenetic tree and venom diversity of centipedes with submitted venom-gland transcriptomes. For each species, the proportion of sequences encoding unique high-molecular-weight venom proteins (HMW, red) and low-molecular-weight venom peptides (LMW, blue) is shown in the first column of pie charts. The proportion of unique sequences contained in each LMW venom peptide family is shown in the second column, with the numbers corresponding to scoloptoxin family (SLPTX). For *T. longicornis*, the scutigerotoxin family (SCUTX) is also noted. The transcriptomes of *S. viridis* [31] and *S. subspinipes mutilans* [32] are not included because of the low number of sequences or selection for short toxin-encoding sequences, respectively.

Until very recently, the toxin arsenals of centipedes remained almost completely unstudied [2]. A few non-peptidic venom components had been described, including 5-hydroxytryptamine (5-HT or serotonin) and histamine [21,22]. However, the large majority of proteinaceous venom components remained mostly undescribed. The novelty of centipede venoms was apparent from early studies of their cardiotoxic and neurotoxic properties, where the responsible venom components were identified as being of surprisingly high molecular weight [23,24]. The prevalence of hitherto undescribed toxin types was also confirmed by *N*-terminal sequencing; of 24 proteins from two species of *Scolopendra* only two CAP [CRiSP (cysteine rich proteins), Allergen (Ag-5), and Pathogenesis-related (PR-1)] proteins were identified [25]. Improvements in sequencing and mass spectrometry platforms have recently enabled more detailed insights into the composition, evolution, and putative mode of action of centipede venoms. Although the taxonomical range of species examined is currently limited to members of the scolopendromorph family Scolopendridae as well as a single scutigeromorph species, these more recent studies confirm that centipede venoms are a rich and diverse source of novel toxins and structural scaffolds (Table 1, Figure 2).

### 3.1. Molecular and Pharmacological Diversity—Enzymes

Mohamed and co-workers [21] were the first to show enzymatic activity in centipede venom, namely phosphatase and esterase activity from the venom of *Scolopendra morsitans*. Since then, 11 types of enzymes have been described from the venoms of Scolopendromorpha and Scutigeromorpha. Some of these have been shown by proteomic analyses to be abundant venom components, indicating that enzymes generally form an important component of centipede venoms [2,26,27,28,29]. Although most centipedes have well developed mandibles that are used for mastication of solid food prior to ingestion [30], the substantial enzymatic component of their venom suggest that it may contribute to extra-oral digestion of prey.

#### 3.1.1. Metalloproteases

Both activity- and sequence-based investigations have revealed that metalloproteases are important components of centipede venoms [27,29]. Transcriptomic and proteomic analyses of the venom proteome of *Thereuopoda* *longicornis* (Scutigeromorpha, Scutigeridae) revealed that astacin-like metalloendoproteases (MEROPS family M12, subfamily A) accounted for ~10% of venom proteins identified [29]. Similarly, analysis of venom by 2D PAGE revealed that proteins with weak sequence homology to blastula protease 10, an M12A member from sea urchin (UniProt: P42674, *E*-value 0.001), were abundant in scolopendrid species included in the same study. This suggests that metalloproteases in scolopendrid venoms could be derived members of the M12A subfamily, although proteolytic activity should be verified to confirm this. While no putative metalloproteases were reported from the venoms of *Scolopendra viridis* or *Scolopendra subspinipes dehaani* [26,31], this may be due to the limitations of the analytical approaches taken. For example, a search against the full set of published centipede-venom protein sequences reveals an EST (NCBI accession number JZ574148) that is highly similar to members of the scolopendrid putative M12A family (lowest *E*-value 3 × 10^−72^, to GASH01000091). Moreover, conducting the same search using the tryptic fragments from spot 2 from the 2D-PAGE of *S. viridis* (Table 7 in ref. [29]) reveals that this protein is actually a member of the same protein family. Hence, M12A proteases are probably a plesiotypic characteristic of centipede venoms.

**Table 1:** Centipede toxin families described to date. Where cysteine patterns are shown, “–” indicates unspecified loop length while “x” signifies a single residue.

Family name	Type		Function	Earliest known recruitment
*Enzymes*				
Protease M12A	Zinc metalloendopeptidase		Unknown, potential spreading factor	Basal
Protease S1	Serine protease		Potentially involved in activation of toxins	Basal
Protease S8	Serine protease		Potentially involved in activation of toxins	Scolopendridae
γ-GT	γ-Glutamyltransferase		Platelet aggregating activity, hemolytic to mouse and rabbit hemocytes	Basal
Chitinase	Glycoside hydrolase family 18		Unknown	Scolopendridae
Lysozyme C	Glycoside hydrolase family 22		Potential antimicrobial component	Scolopendridae
Hyaluronidase	Glycoside hydrolase family 56		Degrades glycosaminoglycans, potentially facilitating the spread of venom components	Scolopendridae
GDH	Glucose dehydrogenase		Unknown	Basal
Carboxylesterase	Type B carboxylesterase		Unknown	Basal
CentiPAD	Peptidylarginine deiminase		Venom activity unknown; catalyses deamination of the guanidine group of arginine residues, potentially involved in post-translational modification of toxins	*Thereuopoda longicornis*
ScolPLA_2_	Phospholipase type A_2_		Venom activity unknown; venom PLA_2_ can be myotoxic, inflammatory, and neurotoxic	Scolopendridae
*Non-enzymatic proteins*				
β-PFTx	β-Pore-forming toxin		Potentially cytotoxic via formation of polymeric pore structures in cell membranes	Basal
CentiCAP1	CAP protein		Unknown	Basal
CentiCAP2	CAP protein		Ca_V_ channel antagonist (KC144967); Trypsin inhibitor (KC144061)	Scolopendridae
CentiCAP3	CAP protein		Unknown	*Scolopendra morsitans*
LDLA protein	LDLA-repeat domain containing protein		Unknown	Basal
Cystatin	Cystatin		Potential protease inhibitor	*Ethmostigmus rubripes*
Transferrin	Transferrin		Potential antimicrobial component	Basal
DUF3472	Protein containing a domain of unknown function type 3472		Unknown	Scolopendridae
DUF1397	Protein containing a domain of unknown function type 1397		Unknown	*Thereuopoda longicornis*
*Completely uncharacterized proteins*				
Family 1	Unknown		Unknown	Scolopendridae
Family 2	Unknown		Unknown	*Scolopendra morsitans*
Family 3	Unknown		Unknown	Scolopendrinae
Family 4	Unknown		Unknown	*Thereuopoda longicornis*
Family 5	Similar to hypothetical protein from *Drosophila mojavensis* (XP_002005038.1, BLAST *E*-value 4.42E-4)		Unknown	Scolopendridae
Family 6	Unknown		Unknown	Scolopendridae
Family 7	Similar to hypothetical protein from *Chthionobacter flavus* (EDY20616.1, BLAST *E*-value 6.13E-7)		Unknown	*Scolopendra morsitans*
Family 8	Unknown		Unknown	*Thereuopoda longicornis*
Family 9	Unknown		Unknown	*Scolopendra morsitans*
Family 10	Unknown		Unknown	*Scolopendra morsitans*
Family 11	Unknown		Unknown	*Scolopendra* spp.
*Peptides*				
SCUTX 1	2 cysteines	C–C	Unknown (e.g., GASR01000100)	*Thereuopoda longicornis*
SCUTX 2	8 cysteines, includes SLPTX family 27	C–C–C–CC–CC	Unknown (e.g., GASR01000101; JZ722897–9)	Basal
SCUTX 3	Proline-rich linear peptides		Unknown (e.g., GASR01000107)	*Thereuopoda longicornis*
SLPTX 1	6 cysteines and a type 2 chitin-binding domain	C–C–C–C–C–C	Unknown (e.g., GASI01000092)	Basal
SLPTX 2	Defensin-like with 6 cysteines	C–C–C–C–CxC	Unknown (e.g., GASI01000163)	*Ethmostigmus rubripes*
SLPTX 3	Helical peptides with 6 cysteines	C–C–C–CC–C	Unknown; K_V_ antagonist (JN646114); **Na_V_ channel antagonist (UniProt: PODL36)**	*Scolopendra* spp.
SLPTX 4	4 cysteines; transcripts may encode additional linear peptides upstream of cysteine-rich peptide	C–C–C–C	Unknown; K_V_ channel antagonist (KC144226); putative synergistic mode of action for peptides encoded by multidomain transcripts (e.g., U-SLPTX_4_-Er1.1 and U-SLPTX_4_-Er1.2 from KF130724).	Scolopendridae
SLPTX 5	5–11 cysteines	C–C–C–C–C–C–C–C–C–C–C	Unknown; Ca_V_ channel agonist (JN646117)	Scolopendrinae
SLPTX 6	4 cysteines	CxC–CxC	Unknown (e.g., GASH01000180)	*Scolopendra morsitans*
SLPTX 7	Putative ICK fold with 6 cysteines	C–C–C–C–CC	K_V_ channel antagonist (JN646115)	*Scolopendra subspinipes*
SLPTX 8	Multiple linear peptides encoded by the same transcript, sometimes upstream of cysteine-rich peptides with 6 cysteines	C–C–C–CCC	Unknown (e.g., KF130762, JZ722863); putative synergistic mode of action (e.g., U-SLPTX_8_-Er5.1a and U-SLPTX_8_-Er5.2a from KF130754)	Scolopendridae
SLPTX 9	6–8 cysteines; transcripts may encode additional linear peptides downstream of cysteine-rich peptide	C–CxC–C–C–C	Unknown; putative synergistic mode of action for peptides encoded by multidomain transcripts (e.g., U-SLPTX_9_-Er4.1a and U-SLPTX_9_-Er4.2a from KF130739)	Scolopendridae
SLPTX 10	6 cysteines	C–C–C–CC–C	Unknown; K_V_ channel antagonist (KC144849); Ca_V_ channel antagonist (KC144448)	Scolopendridae
SLPTX 11	4–18 cysteines	C–C–CxC–C–C–C–CxC–C–C–C–CxC–C–C (e.g., KC144104); C–CxC–C (e.g., JN646116)	Unknown; K_V_ channel antagonists (e.g., JN646116, KC144104); Anticoagulant (KC144430)	*Scolopendra* spp.
SLPTX 12	7 cysteines	C–C–CxC–CxC–C	Unknown (e.g., GASI01000120)	Scolopendridae
SLPTX 13	8 cysteines	C–C–CC–C–C–CxC	Unknown; Ca_V_ channel antagonists (JN646118)	Scolopendridae
SLPTX 14	8 cysteines	C–C–C–CC–CxCxC	Unknown (e.g., GASI01000125)	Scolopendridae
SLPTX 15	4–6 cysteines	C–C–CxC	Unknown; K_V_ channel antagonists (KC144556); Na_V_ antagonists (KC144793); Ca_V_ channel antagonists (KC145039)	Scolopendridae
SLPTX 16	Von Willebrand factor type C; peptides with 3–9 but predominantly 8 cysteines	C–C–C–C–C–CC–C; C–C–C–C–C–CCC–C (e.g., GASI01000127)	Unknown (e.g., GASI01000135)	Scolopendridae
SLPTX 17	Predominantly 8 cysteines	C–C–C–CC–C–C–C	Unknown (e.g., GASI01000156)	*Ethmostigmus rubripes*
SLPTX 18	Colipase-like peptides with 10 cysteines	C–C–CC–C–C–CxC–C–C	Putative colipase, same superfamily as AVIT-toxins which induce smooth muscle contraction and hyperalgesia (GASI01000011)	*Ethmostigmus rubripes*
SLPTX 19	12 cysteines	C–C–C–C–CC–C–C–C–C–CC	Putative carboxypeptidase inhibitor (e.g., GASH01000169)	Basal
SLPTX 20	6 cysteines	C–C–C–C–CC	Unknown (e.g., GASH01000170)	Scolopendrinae
SLPTX 21	Linear diuretic hormone-like peptide		Unknown (e.g., GASH01000171)	*Scolopendra morsitans*
SLPTX 22	Linear hypertrehalosaemic hormone-like peptide		Unknown (e.g., GASI01000170)	Scolopendridae
SLPTX 23	Linear peptide		Unknown (e.g., GASH01000173)	*Ethmostigmus rubripes*
SLPTX 24	Linear peptide		Unknown (e.g., GASH01000177)	*Ethmostigmus rubripes*
SLPTX 25	Linear peptide		Unknown (e.g., GASH01000182)	*Ethmostigmus rubripes*
SLPTX 26	7 cysteines	C–C–C–C–C–CC	Unknown (JZ722896)	*Scolopendra subspinipes mutilans* [32]
SLPTX 28	3 cysteines	C–CC	Unknown (JZ722900)	*Scolopendra subspinipes mutilans* [32]

Although the M12 family has been recruited into the venoms of most groups of venomous animals [33,34,35,36,37,38,39,40], the majority of these are members of subfamily M12B. Exceptions include metalloproteases in the venom from spiders of the genus *Loxosceles*, and from the nematocysts of the sea anemone *Nematostella vectensis*, which belong to M12A [35,41]. Many members of the M12A subfamily cleave matrix proteins and could thereby facilitate the spread of other centipede-venom components [42], a function that has also been suggested for spider-venom proteases [43]. In addition, venom metalloproteases are often involved in skin damage, oedema, blister formation, myonecrosis and inflammation, and this is consistent with several of the recurrent symptoms associated with centipede stings (see Supplementary Table in Ref. [2]).

#### 3.1.2. Serine Proteases

In addition to metalloproteases, serine protease activity has been demonstrated from scolopendrid centipede venom [27]. Supporting this, both S1 and S8 type protease transcripts and venom proteins have been identified from both subfamilies of Scolopendridae (Otostigminae and Scolopendrinae) [29,31,44]. While venom S8 proteases appear to be unique to centipedes, S1 proteases have been widely recruited into animal venoms where they are involved in a range of functions, including vasodilation, smooth muscle contraction, anticoagulation and immunosuppression [39,40,45,46,47].

However, as evident from proteomic analyses, S1 and S8 proteases are not particularly abundant in centipede venoms, and proteolytic activity can be virtually abolished by incubating venom with the metal chelator 1,10-phenanthroline [26,27,29]. Metalloproteases therefore appear to be the dominant form of proteases in centipede venom, and serine proteases may instead play a role in toxin processing [48,49]. This suggests that toxins are activated during storage subsequent to release into the extracellular space, upon venom expulsion, or even both. Consistent with this hypothesis, Undheim and co-workers found that mature toxins encoded by multi-toxin transcripts are present in the venom gland [50]. It also raises the possibility that venom obtained by electrostimulation may contain unprocessed or partially processed toxins due to the involuntary secretion of venom, perhaps explaining the finding by Rates and co-workers [25] that the same toxin was present with and without a 10-residue *N*-terminal tail.

#### 3.1.3. γ-Glutamyl Transpeptidase

γ-Glutamyl transpeptidases (GGTs) are enzymes involved in regulation of oxidative stress and xenobiotic detoxification [51]. GGT was previously reported from the venom of parasitoid wasps, where it is proposed to induce apoptosis of host ovaries via oxidative stress [36,52]. Although it appears not to be present in other centipede venoms, transcriptomic and proteomic analyses show that GGT is both highly expressed and abundant in scolopendrine (Scolopendridae) venoms [29]. Centipede-venom GGT induces aggregation of human platelets and hemolysis of red blood cells from mice and rabbits but not humans [26]. However, targeting vertebrate hemostasis is unlikely to be the primary function of centipede-venom GGT due to the small body size of many scolopendrid species in which GGT forms a major venom component (e.g., *Cormocephalus*) [29]. Nevertheless, the abundance of GGT suggests that it is an important constituent of scolopendrine venoms that was probably recruited into the venom subsequent to the split between the two scolopendrid subfamilies approximately 230 mya [53]. 

#### 3.1.4. Glycoside Hydrolases

Members of the glycoside hydrolase (GH) superfamily hydrolyze the glycosidic bond between carbohydrates or between a carbohydrate and a non-carbohydrate moiety. Three GH families have been found in venoms from Scolopendridae, namely chitinase (GH family 18; *Cormocephalus*), lysozyme (GH family 22; *Scolopendra*), and hyaluronidase (GH family 56; *Scolopendra*, *Ethmostigmus*, and *Otostigmus*) [27,29,31]. While chitinases are found in several venoms and could perhaps aid in digestion of arthropod prey [54,55,56,57], lysozyme hydrolyses β-1,4-links between *N*-acetylmuramic acid and *N*-acetyl-d-glucosamine in the peptidoglycan of bacterial cell walls and could therefore act as an antibacterial agent [46,58]. Hyaluronidases hydrolyse non-sulfated glycosaminoglycans that are widely distributed in connective, epithelial, and neural tissues as well as extracellular matrix, and hence are often regarded as “spreading factors” that increase the pathological impact of other venom components [45,59,60,61,62,63]. 

#### 3.1.5. Phospholipase A_2_

PLA_2_ are found in a very wide range of animal venoms, where they display a diverse array of catalytic and derived non-catalytic activities [45,64]. In centipedes, however, PLA_2_ activity has so far been found only in scolopendrid venoms [26,27,28,29]. Phylogenetic analysis of centipede-venom PLA_2_ revealed that they form a monophyletic group and thus originate from a single recruitment event [29]. According to the available data, this probably occurred prior to the split between the two scolopendrid subfamilies approximately 230 mya but subsequent to the split from Cryptopidae ~200 mya [3]. Centipede-venom PLA_2_ are also unique in that they form a sister-clade to Group X-related PLA_2_, unlike any venom or invertebrate PLA_2_ described to date [29,45]. 

Although PLA_2_ was recruited into the venom of a scolopendrid ancestor, not all centipede venoms have PLA_2_ activity. PLA_2_ hydrolyse glycerophospholipids at the *sn-2* position to release lysophospholipids and fatty acids such as arachidonic acid. However, neofunctionalisation of snake-venom PLA_2_ often removes the ability to catalyse this reaction [45,65], and this may also be the case for PLA_2_ in scolopendrid venoms. For example, ScolPLA from the venom of *Scolopendra viridi* has a high level of PLA_2_ activity, but no PLA_2_ activity was detected in venom from a *Scolopendra* sp. collected in the same locality [28,66]. Neofunctionalisation might also explain the low PLA_2_ activity found in the venoms of *Otostigmus pradoi* and *Scolopendra viridicornis*, although the abundance of PLA_2_ in these venom was not determined [27]. In some cases, PLA_2_ appears to have been secondarily lost, such as in *Cormocephalus westwoodi* where no PLA_2_ was detected in the venom proteome and only transcripts containing numerous stop codons were found in the venom-gland transcriptome [29].

#### 3.1.6. Other Enzymes

In addition to the abundant and commonly recruited enzymes described above, a number of other less abundant or unusual enzymes have been found in centipede venoms. Among these is glucose-6-phosphate dehydrogenase (EC 1.1.1.49). The role of this enzyme in venoms remains to be determined, but proteomic data indicate that it is relatively abundant in scolopendrid venoms and is potentially present in scutigerid venoms [29]. Glucose-6-phosphate dehydrogenase catalyses the first step of the pentose phosphate pathway [67] but this ancestral activity is unlikely to contribute to toxin processing or venom toxicity. Thus, given its abundance, venom glucose-6-phosphate dehydrogenase likely represents a case of protein neofunctionalisation.

Perhaps the most novel enzyme found in centipede venom is centipede peptidyl arginine deiminase (centiPAD). This enzyme has not been reported from any other animal venom, but several isoforms were detected in venom from the scutigerid *T. longicornis* [29]. CentiPADs are distinct from mammalian PADs but similar to *Porphyromonas*-type peptidyl arginine deiminase, which catalyses deamination of the guanidino group on *C*-terminal arginine residues to yield ammonia and a citrullinated residue [68]. The function of CentiPADS in the venom or venom gland remains to be determined, but they might be involved in posttranslational modification of toxin arginine residues.

Judging from proteomic data, esterases are among the least abundant enzymes in centipede venoms that are commonly found in other animal venoms. Esterases have been reported from the venoms of diverse taxa such as spiders [69,70], snakes [71,72] and octopus [73], and in fact the first enzymatic activity reported from centipede venom was esterase activity noted in venom-gland extracts of *Scolopendra morsitans* [21]. This activity is likely due to type B carboxyl esterase, which was subsequently found in the venom of *Cormocephalus westwoodi* and identified in venom-gland transcriptomes from the scolopendrids *S. morsitans* and *S. alternans*, and the scutigerid *T. longicornis* [29]. Venom carboxyl esterases have been proposed to play a part in the release of endogenous purines during envenomation, which then act as “multitoxins” that cause a multitude of pharmacological effects including immobilization through hypotension [74,75]. However, the function of centipede-venom esterases remains to be determined. 

### 3.2. Molecular and Pharmacological Diversity—Non-Enzymatic Proteins

#### 3.2.1. Centipede β-Pore-Forming Toxins

Among the proteomically most abundant and most highly expressed proteins in centipede venoms are putative β-pore-forming toxins (β-PFTx) [29]. These toxins were probably recruited into an early common centipede ancestor more than 430 mya and have subsequently undergone extensive radiation [3,29]. β-PFTx contain a pore-forming domain termed the β-complex domain. This structural domain, which is directly involved in pore formation, is characteristic of the aerolysin-like β-pore-forming toxin superfamily. Pore formation occurs via assembly of toxin monomers to form a β-barrel, which then undergoes a conformational change and inserts into the membrane to form a transmembrane pore [48]. Oligomerization of β-PFTx monomers is mediated by binding of the toxins to various cell-surface receptors via additional toxin domains; thus, the diversity of centipede β-PFTx might enable them to target a wide variety of cell types and tissues and assert multiple toxinological functions. Aerolysin requires proteolytic activation in order to oligomerize into a pore-forming heptamer, and this could be carried out by a number of proteases including S1 and S8 types [48]. Thus, one possible function of centipede-venom serine proteases might be activation of β-PFTx upon envenomation. 

Although the pore-forming properties of centipede β-PFTx have yet to be directly demonstrated, they might be at least partly responsible for the cytolytic activity of centipede venoms [27,76]. Pore-forming activity by β-PFTx might also explain the report that an 80-kDa centipede-venom protein induced an increased leak current in giant axons of the American cockroach *Periplaneta americana* [24]. β-PFTx might also contribute to the myotoxic and oedematogenic activities of centipede venoms that are evident in the symptoms associated with human envenomations [2,27].

#### 3.2.2. CAP Proteins

CAP proteins have been widely recruited into animal venoms, where they can function as ion channel modulators, vasodilators, myotoxins, or even proteases [41,45,62,77]. CAP proteins constitute a major component of centipede venom, and phylogenetic analysis indicates that they have been recruited into centipede venom on three separate occasions: once in an early ancestor over 430 mya (Type 1; centiCAP1), once in a scolopendrid ancestor at least 200 mya (Type 2; centiCAP2), and once within the past 100 million years in the genus *Scolopendra* (Type 3; centiCAP3) [3,29,53]. CentiCAP1 have only been found in the scutigerid *T. longicornis* and the scolopendrid *E. rubripes*, while centiCAP3 have been reported only in *S. morsitans* [29]. CentiCAP2 are the dominant form in Scolopendrinae, where they have diversified into multiple subtypes and undergone neofunctionalisat-ion to include inhibitors of trypsin and voltage-gated calcium (Ca_V_) channels [25,26,29,31]. The activities of centiCAP1 and centiCAP3, and most centiCAP2, remain to be determined but they might be of clinical relevance by virtue of their high abundance. CAP proteins are among the principal allergens in vespid and fire ant (*Solenopsis* spp.) venoms [78], and therefore the relatively frequent allergic reactions observed after centipede envenomation (see Supplementary Table in Ref. [2]) might be due at least in part to the abundant centiCAPs.

#### 3.2.3. LDLA Domain-Containing Proteins

In addition to β-PFTx and centiCAPs, proteomic analyses show that novel proteins containing a low-density lipoprotein receptor Class A repeat (LDLA) domain are a major constituent of centipede venoms [26,29]. The LDLA structural domain, which comprises a β-hairpin motif followed by a series of β turns, is present in a wide variety of proteins [79]. LDLA-proteins were recruited in an early centipede ancestor at least 430 mya, and they have subsequently undergone substantial diversification [29]. LDLA proteins have not been reported from any other venom, and the function of LDLA-containing centipede-venom proteins remains to be determined. Nevertheless, the abundance and diversification of centipede-venom LDLAs suggests that they are important components of the venom.

#### 3.2.4. Other Non-Enzymatic Proteins

In addition to the abundant protein families described above, centipede venoms contain a number of other proteins that are probably non-enzymatic, including transferrin and cystatin. Transferrin has been identified in venom-gland transcriptomes from both scolopendrid subfamilies, the scutigerid *T. longicornis*, and the venom of *E. rubripes* and *S. morsitans* [29]. Centipede-venom transferrins may have an antibacterial function since invertebrate transferrins have been implicated in pathways involved in the reaction to secondary infections [80]. 

Two isoforms of cystatin were identified in venom from the scolopendrid *E. rubripes* [29]. Cystatins are potent inhibitors of papain family cysteine proteases, although they have acquired new functions when recruited into reptile venom, *Lonomia* caterpillar bristles, and the saliva of ticks and mosquitoes [45,81]. However, both of the centipede-venom isoforms contained the characteristic peptidase-interacting sequence Gln-X_aa_-Val-X_aa_-Gly as well as the cystatin type-1 like Pro-Gly pair, suggesting that they have retained their ancestral function as peptidase inhibitors [82].

The majority of non-enzymatic protein families found in centipede venoms appear to be novel; they cannot be assigned a putative function or to a known protein family. Undheim and co-workers [29] identified eleven protein families in venoms from three scolopendrids and one scutigerid, as well as two protein families containing only domains of unknown function (DUF). One of these domains (DUF 1397) was identified only in scutigerid venom while the other (DUF 3472) was only found in the scolopendrid venoms and transcriptomes.

### 3.3. Molecular and Pharmacological Diversity—Peptides

Low molecular weight (LMW) peptides (*i.e.*, peptides <10 kDa) form an important component of most centipede venoms studied to date. Venom peptides are of significant interest from a biodiscovery perspective and hence they are likely to attract the most attention from toxinologists. Rapid growth in the number of described centipede-venom peptides has necessitated development of a systematic nomenclature for naming these toxins. We recently proposed a rational nomenclature in which peptides are named according to their first described pharmacological activity, the phylogenetically determined peptide family, genus and species from which the peptide was isolated, peptide number, and isoform [29]. Pharmacological activity is denoted by a Greek letter as proposed for spiders [83], while the peptide family name takes the form of a capitalized abbreviation of the peptide group followed by a subscripted peptide family number. Finally a two- or three-letter species code is provided followed by the peptide number and isoform. Thus, for example, µ-SLPTX_15_-Ssd1a (SSD800; KC144793) is the first toxin and isoform (1a) that modulates the activity of voltage-gated sodium channels (µ) from *Scolopendra subspinipes dehaani* in scoloptoxin family 15 (SLPTX_15_). This systematic nomenclature readily conveys both pharmacological and phylogenetic information, thereby providing a classification system that should minimize confusion and redundancy.

#### 3.3.1. Molecular Diversity of Centipede Venom Peptides

Peptides stabilized by one or more intramolecular disulfide bonds are of particular interest from a drug and insecticide discovery perspective due to their stability and inherent plasticity to amino acid mutations. Not surprisingly, these are the same properties that make disulfide-rich peptides amenable to toxin recruitment and neofunctionalisation [45]. As a result, disulfide-rich peptides make up a large fraction of the toxin arsenal in many venomous animals, including spiders, scorpions and marine cone snails [43,84,85,86].

In centipedes, disulfide-rich peptides constitute the bulk of venom-peptide abundance and diversity (Table 1). Although not as abundant in centipede venoms as they are in spider venoms [87], mass spectrometry investigations into the LMW composition of scolopendrid venoms have shown that they contain a relatively large number of peptides. Fifty-three and 50 unique masses <10 kDa were detected in the venoms of *Scolopendra viridicornis* nigra and *S. angulata*, respectively [25], while 40 unique LMW masses were identified in venom from *Scolopendra viridis* [31]. Peptide masses display a bimodal distribution in *S. viridis*, with the majority between 4–5 kDa and 8–9 kDa, whereas masses have a more Gaussian distribution in both *S. v. nigra* and *S. angulata*, with most peptides having a mass of 4–6 kDa [25,31]. The unimodal distribution of masses in these latter species fits better with the distribution of masses predicted from transcriptomic data [26,29].

Although the richness (*i.e.*, number of masses detected) of scolopendrid venom peptides does not match that of spiders, the diversity is nevertheless astounding. To date, 30 phylogenetically distinct families have been described from eight species, with 24 of these families being cysteine-rich [25,26,29,31,32,88]. These cysteine-rich SLPTX families are structurally diverse, with molecular weights varying between 3 and 20 kDa and the number of disulfide bonds ranging from 2 to 9. This exceptional structural diversity is exemplified by the SLPTX_11_ family, which to date has only been found in the genus *Scolopendra* [26,29,31,89]. The first described member of SLPTX_11_ was the 8-kDa voltage-gated potassium (K_V_) channel inhibitor κ-SLPTX_11_-Ssm3a [89]. However, phylogenetic analysis revealed that κ-SLPTX_11_-Ssm3a is in fact a truncated form of a family dominated by cysteine-rich proteins with molecular weights of ~20 kDa [29]. Several other independent truncation events, as well as one insertion event, have also occurred in SLPTX_11_ with the result that its members range in size from 6.7 to 25.6 kDa and contain between 6 and 19 cysteine residues. 

Such structural diversification by truncation and loss or acquisition of additional disulfide bonds is not exclusive to SLPTX_11_. These events have also occurred in the SLPTX_16_ family, which is found in all scolopendrids and comprises toxins ranging in size from 7.4 to 13.6 kDa. Although most of these toxins contain an even number of cysteine residues (8), there are also members containing 3, 5, and 9 cysteines that may form dimeric or higher-order complexes [29]. Scolopendrid centipede venoms are thus somewhat unusual in that a single toxin family may span a wide molecular weight range, including both low (<10 kDa) and high (>10 kDa) molecular weight venom components. A single transcript may also encode both linear and disulfide-rich peptides, as has been shown to be the case in SLPTX families 4, 8, and 9 [50]. Furthermore, while the inhibitor cystine knot and cysteine-stabilized α/β defensin folds that dominate spider and scorpion venoms, respectively, are present, these appear to constitute only a minor part of scolopendrid venoms [29].

However, the diversity of LMW venom peptides found in scolopendrid venoms appears not to be representative of all centipedes. Although the taxonomic coverage of centipede venoms that have been studied is currently very poor, there are striking differences in the abundance and diversity of cysteine-rich peptides between scutigerid and scolopendrid venoms (Figure 2). In the only study to include a non-scolopendrid centipede, three cysteine-rich peptide families were identified in the venom of *T. longicornis* [29]. Of these, scutigerotoxin family 1 (SCUTX_1_) contains a single isoform with one disulfide bond, SCUTX_2_ contains six isoforms with two to eight disulfide bonds, and scoloptoxin family 1 (SLPTX_1_) four isoforms with three disulfide bonds. 

The latter of these cysteine-rich peptide families, SLPTX_1_, is particularly interesting in an evolutionary sense due to the hypothesized epidermal origin of the centipede venom gland. Members of SLPTX_1_ are characterized by the presence of a single type 2 chitin-binding domain (CB_2_ domain; InterPro accession IPR002557) and they are also found in the venoms and venom-gland transcriptomes of both subfamilies of Scolopendridae [29,31]. In addition, homologous sequences containing three CB_2_ domains are expressed by epidermal cells in *E. rubripes*, suggesting an epidermal origin of SLPTX_1_ [29]. Thus, SLPTX_1_ probably represents one of the first cysteine-rich peptides recruited into the venom of an early venomous common centipede ancestor over 430 mya [3].

#### 3.3.2. Pharmacological Diversity of Centipede Venom Peptides

In spiders, a large number of venom peptides have evolved to target the nervous system of their prey by modulating the activity of ion channels, often with high potency and specificity [43]. While ion channel modulating activities have been described for centipede-venom peptides, only one study has so far identified potent insecticidal peptides [89]. Although crude centipede venom is lethal to both insects and crustaceans, fractionation by reverse-phase HPLC appears to abolish these properties in several scolopendrid venoms [25,31]. While this could be explained by denaturation during fractionation [25,66], the exceptional stability of most venom peptides suggests that a more plausible explanation might be synergistic modes of toxin action [31]. Synergism has been hypothesized for two scolopendrid toxin families where multiple toxins are expressed on the same transcript and the evolutionary selection regime suggests that the activity of each mature toxin is dependent upon the other [50].

Despite the lack of lethal activity of many centipede-venom peptides, several ion channel modulating peptides have been described from the venom of *S. subspinipes* (Table 1) primarily based on screening against ionic currents in rat dorsal root ganglion (DRG) neurons. The first of these were several modulators of voltage-gated calcium, potassium, and sodium channels (Ca_V_, K_V_, Na_V_, respectively) described from the venom of *S. subspinipes mutilans* [89]. Among these was a 3763 Da, two disulfide, Na_V_ channel inhibitor named µ-SLPTX_3_-Ssm1a that inhibited tetrodotoxin-sensitive Na_V_ currents with an IC_50_ of 9 nM. Interestingly, the *N*-terminal sequence of µ-SLPTX_3_-Ssm1a is almost identical to that of another more recently described Na_V_ inhibitor, µ-SLPTX_3_-Ssm6a, which has an IC_50_ of 23 nM but a mass of 5318 Da and three disulfide bonds [90]. A member of SLPTX family 15 has also been described that inhibited Na_V_ currents in rat DRG neurons, although potency was not quantified [26]. Weak inhibition of human Na_V_ subtypes 1.2 and 1.6 were also detected in the crude venom of *S. viridis* suggesting that this venom also contains Na_V_ inhibiting toxins [31].

Both Ca_V_ agonist and antagonist activities have also been described from two subspecies of *S. subspinipes* [26,89]. Interestingly, the only agonist described, ω-SLPTX_5_-Ssm1a, is also the only venom-derived Ca_V_ agonist described to date [89]. Although it is not particularly potent, with a micromolar EC_50_, ω-SLPTX_5_-Ssm1a is unusual in that it contains an odd number of cysteine residues. It does not appear to be insecticidal, suggesting it either acts synergistically with other venom components or plays a non-insecticidal role in the venom. Nevertheless, the unique activity of ω-SLPTX_5_-Ssm1a suggests it could prove to be a useful pharmacological tool.

The majority of Ca_V_ modulators described from venoms of *S. subspinipes* are antagonists. These toxins are structurally diverse and include members of SLPX families 10, 13, and 15, which all have a molecular weight of about 6 kDa but contain different cysteine scaffolds [26,29,89]. Not much is known about the pharmacological properties these peptides. Selectivity has not been investigated, and activity has only been quantified for ω-SLPTX_13_-Ssm2a, which has an EC_50_ of ~1.6 µM for inhibition of Ca_V_ channel currents in rat DRG neurons [89]. This relatively low potency is probably similar to ω-SLPTX_15_-Ssd1a (SSD1052; KC135039), which at 10 nM inhibited Ca_V_ currents in rat DRG neurons by about 8.6% [26]. Interestingly, this latter peptide belongs to family SLPTX_15_, which is a prime example of functional radiation of centipede-venom peptides since it contains not only Ca_V_ channel antagonists but also inhibitors of Na_V_ and K_V_ currents in DRG neurons [26,29].

Although centipede venoms contain both Na_V_ and Ca_V_ channel modulators, modulation of K_V_ channels may be an even more dominant pharmacology. Of eight scoloptoxin families containing peptides with characterized activity, six contain at least one K_V_ inhibitor [29]. Needless to say, centipede-venom K_V_ inhibitors include a very diverse set of venom peptides, ranging from the eight-disulfide, 22.5-kDa members of SLPTX_11_ to the three-disulfide, 3.5-kDa κ-SLPTX_7_-Ssm2a [26,89]. The potency and selectivity of these toxins also appears to be quite variable. The most potent K_V_ inhibitor described to date is κ-SLPTX_15_-Ssd1a (SSD559; KC144556), which irreversibly inhibits K^+^ currents in DRG neurons with an IC_50_ of 10 nM [26]. In comparison, κ-SLPTX_11_-Ssm3a has an IC_50_ in the low micromolar range, and it does not fully inhibit peak K^+^ currents in DRG neurons even at 5 µM [89]. However this toxin was a more potent inhibitor of slowly activating rectifier K^+^ currents, which would be complementary to the activity of other peak current inhibiting toxins [89]. 

In addition to ion channel modulators, four antimicrobial peptides have been described from the venom of *S. subspinipes mutilans.* While the sequence of one of these, scolopendrin 1, was not determined, the remaining three are “linear” (*i.e.*, non-disulfide reticulated) peptides [88,91,92]. All potently kill Gram-positive and Gram-negative bacteria as well as fungi, but differ in their additional non-antimicrobial properties. Scolopendrin I, the first antimicrobial centipede-venom peptide to be described, showed no hemolytic or agglutination activity against mouse erythrocytes and may therefore function exclusively as an antimicrobial agent [91]. The unnamed linear peptide described by Kong and co-workers [92] also showed some anticoagulant properties. However, this turned out to be a proteolytic fragment of a member of SLPTX_15_, and it is therefore most likely an artifact resulting from the purification process. In contrast, the two remaining peptides, scolopin-1 and -2, showed moderate hemolytic activity against both human and rabbit red blood cells [88]. More interestingly, however, at ~30 µM scolopin-1 and -2 caused release of histamine from mast cells harvested from the peritoneum of rats. This suggests these peptides are also involved in the general toxic effect of the venom of *S. subspinipes mutilans* through the release of endogenous histamine, which is thought to be the case for antimicrobial peptides from spider venoms [93]. Although neither scolopendrin I nor any of the scolopins have been found in any other scolopendrid species, a number of linear peptides with unknown function have been identified and these peptides may have similar antimicrobial and/or histamine-releasing roles. 

## 4. Clinical Importance of Centipede Stings

Centipedes are notorious for producing painful stings. However, systemic or serious local symptoms are rare, and most stings are left unreported [94,95,96]. A few stings by scolopendromorph centipedes from the families Cryptopidae and Scolopocryptopidae as well as one lithobiomorph centipede have been reported, but the vast majority appear to be caused by members of the Scolopendridae [2]. This skewed statistic may well be due to the less cryptic nature of scolopendrid centipedes, which are commonly encountered foraging at night in warmer climates. However, another explanation could also be that the severity of their stings is greater and therefore more likely reported to medical personnel. Lithobiid centipedes, for example, are common in gardens of suburban Europe, while *Scutigera coleoptrata* (Scutigeromorpha) is aptly named “house centipede” due to its abundance around human dwellings. Despite their ferocious reputation, all centipedes tend to attempt to escape rather than attack, which is reflected in the vast majority of envenomations occurring around the extremities of limbs such as hands and feet (see Supplementary Table in Ref. [2]).

Although their venoms harbor an abundance of potential cytotoxins, proteases, neurotoxins and allergens, centipede stings cannot be generally regarded as life-threatening [2]. There are a few human fatalities attributed to centipede stings, but most of these are without any reported symptoms or cause of death. In the USA, five human fatalities due to centipedes were reported between 1991 and 2001 and only two between 1997 and 2007, although no actual cause of death was presented [97,98]. In comparison, hymenopterans were responsible for 533 and 509 deaths, respectively, over the same time periods. There are also instances where fatalities have been attributed to centipede envenomation despite total lack of evidence for centipede involvement in the mortality, such as the claims in some citations of the case reported by Harada and co-workers [99]. Secondary infections can also result from centipede stings [100,101,102,103,104], and these can in very rare cases lead to serious complications or even death [105]. The only substantiated deaths occurring from centipede envenomation therefore appear to be that of a 7 year-old boy in the Philippines that was stung on the head and died 29 hours later [106] cited in e.g., [107]), a 21 year-old female stung by a centipede in Thailand [95], and an army officer from Mauritius who accidently drank a small centipede and was stung in the back of the throat and probably died by asphyxiation [108].

While centipede envenomations are very rarely fatal, the high abundance of allergen-related proteins in centipede venoms poses a significant risk after envenomation. A relatively high proportion of humans are sensitized to hymenopteran venom allergens, and these people are at risk of experiencing similar reactions to centipede venoms. CentiCAP proteins are among the most abundant proteins in centipede venoms, and these are also among the principal allergens in the venoms of vespids (e.g., yellowjackets, hornets, paperwasps) and formicids (e.g., fire ants) [109]. Centipede venoms also contain a range of known apid (bee) allergens such as PLA_2_, hyaluronidase, and S1 peptidase, although the lack of reactivity to centipede venom by prick test in patients allergic to bee venom suggests that these proteins may not have the same allergenic properties as centiCAPs [110]. Nevertheless, in combination with histamine-releasing peptides such as scolopin-1 and -2, allergenic proteins have the potential to cause histamine-related complications. Reflecting this, administration of anti-histamines has been reported to alleviate symptoms following centipede envenomation [111,112,113].

Most centipede envenomations only result in local symptoms, which often include intense pain and swelling (see Supplementary Table in Ref. [2]). Although the specific mode of action remains to be determined, the abundance of large putative pore-forming proteins (β-PFTx) and metalloproteases may partly explain the prevalence of these symptoms. This might also explain the apparent effectiveness of hot water immersion in alleviating pain and swelling after centipede envenomation, as these proteins appear to be quite labile [24,95,112]. However, application of ice is also reported to be effective at reducing pain, and even comparable to the use of analgesics [112]. Given the diversity of toxin families in centipede venoms, the painful symptoms that usually characterize centipede envenomations are probably due to the actions of several of these and will almost certainly vary with species.

## 5. Conclusions

Despite being among the most ancient extant venomous animals, little is currently known about the evolution, ecology, and molecular and pharmacological diversity of centipede venoms. However, there appears to be significant differences in venom composition, and presumably venom strategies, between different centipede orders. It has been postulated that centipede venoms function via a two-step mechanism where non-peptidic and neurotransmitter-releasing peptide neurotoxins produce a rapid, transient paralysis that is followed by the lethal actions of larger myotoxic and neurotoxic proteins [2]. Although the venom of only a single non-scolopendrid centipede species has so far been examined, it appears that this prediction may be more accurate for non-scolopendrid than scolopendrid centipedes. In contrast to non-scolopendrids, scolopendrids have a rich and highly diverse arsenal of neurotoxic peptides much like those of spiders and scorpions. However, the vast majority of centipede toxins remain functionally uncharacterized, and consequently little is known about the overall mechanism of action of the venom. Fortunately, the development of increasingly sensitive and accurate analytical tools is enabling increased access to venomous species of ever-decreasing size, allowing for greater taxonomic coverage. Moreover, recent publication of the first centipede genome presents new opportunities to gain insight into the genetic mechanisms that underlie the evolution of centipede venom [114]. Finally, the recent recognition of the tremendous molecular diversity of centipede venoms will hopefully generate renewed interest in the venoms of these fascinating arthropods.
